# A novel index, Chinese visceral adiposity index is closely associated with urinary albumin‐creatinine ratio in Chinese community adults, especially in hypertensive or hyperglycemic population: Results from the REACTION study

**DOI:** 10.1111/1753-0407.13336

**Published:** 2022-11-29

**Authors:** Binqi Li, Weiqing Wang, Zhengnan Gao, Xulei Tang, Li Yan, Qin Wan, Zuojie Luo, Guijun Qin, Lulu Chen, Guang Ning, Yiming Mu

**Affiliations:** ^1^ School of Medicine Nankai University Tianjin China; ^2^ Department of Endocrinology First Medical Center of PLA General Hospital Beijing China; ^3^ Shanghai National Research Centre for Endocrine and Metabolic Diseases, State Key Laboratory of Medical Genomics, Shanghai Institute for Endocrine and Metabolic Diseases, Ruijin Hospital Shanghai Jiaotong University School of Medicine Shanghai China; ^4^ Dalian Central Hospital Dalian Liaoning China; ^5^ First Hospital of Lanzhou University Lanzhou Gansu China; ^6^ Zhongshan University Sun Yat‐sen Memorial Hospital Guangzhou Guangdong China; ^7^ Southwest Medical University Affiliated Hospital Luzhou Sichuan China; ^8^ First Affiliated Hospital of Guangxi Medical University Nanning Guangxi China; ^9^ First Affiliated Hospital of Zhengzhou University Zhengzhou Henan China; ^10^ Wuhan Union Hospital, Huazhong University of Science and Technology Wuhan Hubei China

**Keywords:** Chinese Visceral Adiposity Index, diabetic complications, diabetic nephropathy, obesity, urinary albumin to creatinine ratio, 中国人群内脏脂肪指数, 尿白蛋白肌酐比, 肥胖, 糖尿病并发症, 糖尿病肾病

## Abstract

**Background:**

The association between the Chinese Visceral Adiposity Index (CVAI) and urinary albumin to creatinine ratio (UACR) has not been illustrated. The current study aimed to investigate the association between CVAI and UACR and to compare the discriminative power of CVAI, triglyceride, body mass index (BMI), waist circumference (WC), and waist‐to‐hip ratio (WHR) with UACR in the Chinese community population.

**Methods:**

This study included 34 732 participants from the REACTION (Risk Evaluation of cAncers in Chinese diabeTic Individuals) study. Binary logistic regression analyses were performed to detect the association between CVAI, triglyceride, BMI, WC, WHR and UACR.

**Results:**

Binary logistic regression analysis showed that, after adjusting for potential confounders, in women, CVAI (odds ratio [OR]:1.16, 95% confidence interval [CI]: 1.01–1.34) and triglyceride (OR: 1.18, 95% CI: 1.04–1.33) were associated with UACR, whereas BMI, WC, and WHR were not associated with UACR; in men, CVAI (OR: 1.24, 95% CI: 1.02–1.50), WC (OR: 1.21, 95% CI 1.00–1.48), and triglycerides (OR: 1.18, 95% CI 0.97–1.44) were associated with UACR, whereas BMI and WHR were not associated with UACR. Stratified analysis showed that the correlation between CVAI and UACR was stronger in the population with 5.6 ≤ fasting blood glucose (FBG) <7.0 or 7.8 ≤ post‐load blood glucose (PBG) <11.1 mmol/L, FBG ≥7.0 or PBG ≥11.1, systolic blood pressure ≥140 mmHg or diastolic blood pressure ≥90 mmHg.

**Conclusions:**

In the Chinese general population, CVAI and UACR were significantly associated in both genders. At higher CVAI levels, the population with prediabetes, diabetes, and hypertension has a more significant association between CVAI and UACR.

## Graphical abstract

**Highlights**

- This is the first large‐sample, multicenter study of the relationship between Chinese Visceral Adiposity Index (CVAI) and urinary albumin to creatinine ratio (UACR) in Chinese community adults.
- CVAI and UACR were significantly associated in both genders.
- At higher CVAI levels, the population with prediabetes, diabetes, and hypertension has a more significant correlation between CVAI and UACR.

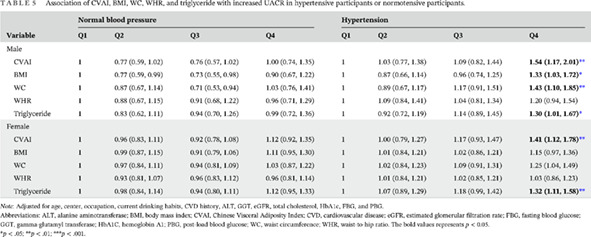

## 摘要

背景:中国人内脏脂肪指数(CVAI)与尿白蛋白肌酐比(UACR)之间的关系尚未阐明。本研究旨在探讨CVAI与UACR之间的关系, 并比较CVAI、三酰甘油、体重指数(BMI)、腰围(WC)、腰臀比(WHR)对中国社区人群UACR的鉴别能力。

方法:本研究包括34732名来自REACTION研究的参与者。采用二元logistic回归分析检测CVAI、三酰甘油、BMI、WC、WHR和UACR之间的相关性。

结果:二元logistic回归分析显示, 在校正潜在混杂因素后, 女性CVAI(优势比[OR]:1.16, 95%CI: 1.01‐1.34)和三酰甘油(OR:1.18,95%CI:1.04‐1.33)与UACR相关, 而BMI、WC、WHR与UACR无关；男性CVAI(OR:1.24,95%CI:1.02‐1.50)、WC(OR:1.21,95%CI:1.00‐1.48)和甘油三酯(OR:1.18,95%CI:0.97‐1.44)与UACR相关, 而BMI、WHR与UACR无关。分层分析表明, 在5.6 mmol/L≤空腹血糖(FBG)<7mmol/L或7.8mmol/L≤餐后2小时血糖(PBG)<11.1mmol/L, FBG≥7.0mmol/L或PBG≥11.1mmol/L,收缩压≥140 mmHg或舒张压≥90 mmHg的人群中, CVAI与UACR的相关性较强。

结论:在中国普通人群中, CVAI和UACR在两性中均显著相关。在较高的CVAI水平下, 糖尿病前期、糖尿病和高血压人群在CVAI和UACR之间具有更显著的相关性。

## 1 INTRODUCTION

Microalbuminuria is the inchoate symbol of diabetic nephropathy and is closely related to cardiovascular disease (CVD),^1^ cardiovascular mortality^2^ and all‐cause mortality.^2^ The LIFE^3^ and PREVEND^2^ investigations found a significant correlation between increasing urine albumin excretion and ischemic CVD morbidity and death. In the general population, the occurrence of microalbuminuria is a sign of diabetes, hypertension, and CVD.^4^, ^5^ A high urinary albumin creatinine ratio (UACR) is not only a symbol of kidney dysfunction but also indicates a higher risk of CVD.^6^, ^7^ Every 3.01 mg/g rise in UACR contributed to a 5.9% increase of CVD incidents.^8^ UACR elevated within the accepted normal range is linked to an increased risk of CVD, as well as an increased risk of the cardiovascular death rate.^9^ Nonetheless, because preventing urinary albumin excretion and renal dysfunction progression is hard in the early phase,^10^ identifying early warning signs for albuminuria is essential.

The correlation between obesity and renal dysfunction was first noted in 1974,^11^ and obesity was found to be highly correlated with albuminuria.^11^ Numerous studies in recent years have demonstrated that visceral adiposity, rather than subcutaneous adiposity or total adiposity, is closely related to an increase in albuminuria.^12^, ^13^, ^14^, ^15^, ^16^ The gold standard for evaluating fat accumulation is magnetic resonance imaging and computed tomography (CT).^17^ However, given the availability, time involved, expense, and radiologic risk, these imaging technologies are inappropriate for regular clinical examine among the general population. Therefore, a practical, user‐friendly, and relatively accurate method for evaluating visceral adiposity mass is urgently required.^18^

Traditional anthropometric measures, such as body mass index (BMI), waist circumference (WC), and waist‐to‐hip ratio (WHR), have the general disadvantage that they do not take into account metabolic indicators and they could not accurately assess visceral adiposity. Chinese Visceral Adiposity Index (CVAI) is a novel visceral fat indication developed specifically for Asian people depending on age, BMI, WC, and metabolic parameters.^19^ A total of 485 participants from Xiamen, China received abdominal CT for visceral adiposity area, then CVAI was established utilizing multivariate linear regression analyses and was further verified in 6495 participants from Shanghai, China that it was highly correlated with visceral adipose and it was more valuable compared to BMI and WC in the assessment of metabolic risks.^19^ Besides, CVAI has been reported to be more significantly related to diabetes,^20^ diabetes complications,^21^ atherosclerosis,^22^, ^23^ polycystic ovary syndrome,^24^ hypothyroidism,^25^ and hypertension^26^ than other obesity indices among the Chinese population, and one study has discovered a close relation between CVAI and diabetes among the Japanese population.^27^ However, the research on the relationship between CVAI and UACR is limited. Therefore, the present study aimed to explore the relationship between CVAI and UACR and compare the discriminative power of CVAI and traditional indicators for UACR in Chinese community adults.

## 2 METHODS

### 2.1 Study subjects

An ongoing cohort study named REACTION (Risk Evaluation of cAncers in Chinese diabeTic Individuals)^28^ designed to research the correlation of diabetes and prediabetes with the risk of cancer in the Chinese population was performed based on the community. The REACTION study is a multicenter, prospective observational study, in which participants have proof of local household registration and be at least 40 years of age, and there is no gender or ethnicity restriction. The present study population came from 7 centers of the REACTION study; 47 808 participants were investigated between March and December 2012. After excluding the corresponding participants according to the exclusion criteria, 34 732 study subjects were enrolled. The exclusion criteria are as follows: (1) suffer from renal disease; (2) use of angiotensin‐converting enzyme inhibitors or angiotensin receptor blocker medicines; (3) use of lipid‐lowering medications; and (4) lack of important information or essential test results.

### 2.2 Questionnaire survey

The participants' essential information was acquired through a standardized questionnaire. The investigators have been professionally trained. The questionnaire broadly covers year of birth, medical history, current medication status, smoking and drinking habits, marriage status, education level, occupation, and other important information.

### 2.3 Physical examination

The height, weight, hip circumference, and WC were measured and documented. The systolic blood pressure (SBP) and diastolic blood pressure (DBP) were measured by the same investigator three times, 5 min apart. The mean values of these three measurements were used in statistical analysis.

### 2.4 Lab measurements

The collection of blood samples was at 8–9 am after 8–10 h of overnight fasting. The following important indicators were measured and documented: (1) total cholesterol, high‐density lipoprotein cholesterol (HDL‐C), low‐density lipoprotein cholesterol (LDL‐C), and triglycerides; (2) fasting blood glucose (FBG), 2 h post‐load blood glucose (PBG), and hemoglobin A1c (HbA1c);(3) serum creatinine (SCr), alanine transferase (ALT), aspartate transferase (AST), and glutamine transferase (GGT); and (4) other biochemical indexes.

Participants without or with diabetes underwent 75 g oral glucose tolerance or 100 g steamed‐bread meal, separately.

Morning urine samples were collected, and urine albumin and creatinine concentrations were determined.

### 2.5 Calculation of UACR, CVAI, BMI, WHR, and estimated glomerular filtration rate (eGFR)

(1) UACR was calculated through dividing the urinary albumin by the urinary creatinine; (2) BMI was expressed in kg/m^2^, BMI = weight (Kg) ÷height^2 (m); (3) WHR = WC÷hip circumference; (4) eGFR was expressed in ml/min per 1.73 m^2^, eGFR = 175 × (SCr in mg/dl) −1.154 × age−0.203 × (0.742 for women) × (1.212 if African American); (5) the CVAI were calculated according to gender as follows:

Males: CVAI = −267.93 + 0.68 × age(y) + 0.03 × BMI (kg/m^2^) + 4.00 × WC (cm) + 22.00 × Lg triglyceride (mmol/L) − 16.32 × HDL‐C (mmol/L).

Females: CVAI = −187.32 + 1.71 × age(y) + 4.32 × BMI (kg/m^2^) + 1.12 × WC (cm) + 39.76 × Lg triglyceride (mmol/L) − 11.66 × HDL‐C (mmol/L).

### 2.6 Definition

CVAI, WC, WHR, BMI, and triglyceride were divided into four groups: the <25% group (the control group), the 25–50% group, the 50–75% group, and the ≥75% group, according to quartile division of the subjects and conducted separately by gender (Table 1). We selected only one lipid, triglyceride, to compare its prediction ability with other obesity indicators for UACR, because previous studies have shown that triglyceride, but not total cholesterol, HDL‐C, or LDL‐C, is significantly associated with UACR.^29^ Increased albuminuria was defined as UACR ≥30 mg/g, indicating renal disfunction.^30^, ^31^ Thus, UACR was divided into two groups: normal group: <30 mg/g and increased albuminuria group: UACR ≥30 mg/g.

**TABLE 1:** Sex‐specific CVAI and traditional indicators in all participants.

Variable	Male	Female
CVAI	116.01 (87.74, 143.27)	100.01 (75.72, 125.85)***
WC	89.50 (83.00, 96.00)	84.00 (78.00, 91.00)***
WHR	0.91 (0.88, 0.95)	0.87 (0.83, 0.92)***
BMI	24.57 (22.52, 26.78)	24.14 (22.01, 26.50)***
Triglyceride	1.40 (0.98, 2.06)	1.36 (0.98, 1.93)***

Abbreviations: BMI, body mass index; CVAI, Chinese Visceral Adiposity Index; WC, waist circumference; WHR, waist‐to‐hip ratio.

*** *p* < .001.

### 2.7 Statistical analysis

SPSS 24.0 (IBM) was used for the statistical analysis. Continuous variables were shown as mean ± SD. Categorical variables were shown numerically (proportionally). Differences in continuous variables between the two groups of UACR were compared using the Kruskal–Wallis test, and categorical variables were analyzed using the chi‐square test. Binary logistic regression was used to test the associations of CVAI, WC, WHR, BMI, and triglyceride with UACR and was conducted separately by gender. Adjustment variables were tested by collinearity diagnosis according to the following criteria: variance inflation factor >10 or tolerance near 0.1; condition index >30; and variance proportions >50%. The adjusted variables had the following criteria: (1) there was a significant difference between the UACR <30 mg/g group and the UACR ≥30 mg/g group in both genders; and (2) there was no collinearity. Model 1 was adjusted for age and center. Model 2: Model 1 + occupation, current drinking habits, and CVD history. Model 3: Model 2 + ALT, GGT, eGFR, and total cholesterol. Model 4: Model 3 + SBP and DBP. Model 5: Model 4 + HbA1c, FBG, and PBG.

After controlling confounders in model 5, the association between independent variables and CVAI was explored within subgroups of blood pressure and blood glucose.

All statistical tests were two sided, and *p* < .05 was considered statistically significant.

## 3 RESULTS

### 3.1 Clinical characteristics of the study population

The current study included 34 732 participants (Table 2), with 10 494(30.2%) males and 24 238(69.8%) females, and their median age (Q1‐Q3) was 57.33(52.18,65.58). Participants (both male and female) with increased UACR had increased age, CVAI, BMI, WC, triglyceride, ALT, GGT, FBG, PBG, HbA1c, SBP, and DBP, as well as decreased HDL‐C and eGFR. The prevalence of CVD events (myocardial infarction, stroke, and coronary heart disease) was higher in both male and female participants with increased UACR.

**TABLE 2:** Characteristics of the study population by UACR category.

Variable	Total	Male		Female	
		UACR <30 mg/g	UACR ≥30 mg/g	UACR <30 mg/g	UACR ≥30 mg/g
Number	34 732	9238	1256	20 666	3572
Age, years	57.33 (52.18, 65.58)	59.17 (53.75, 65.14)	62.47 (55.79, 70.18)***	56.05 (51.35, 61.54)	59.95 (53.80, 68.06)***
CVAI	104.31 (78.70, 131.42)	114.47 (86.19, 141.44)	127.26 (99.49, 155.40)***	97.89 (74.04, 123.14)	114.07 (87.56, 139.72)***
BMI, Kg/m^2^	24.30 (22.15, 26.61)	24.51 (22.49, 26.71)	25.05 (22.86, 27.36)***	24.09 (21.96, 26.44)	24.45 (22.25, 27.02)***
WC, cm	86.00 (80.00, 93.00)	89.00 (83.00, 95.0)	91.00 (85.00, 98.00)***	84.00 (78.00, 90.33)	86.00 (80.00, 93.00)***
WHR	0.89 (0.84, 0.93)	0.91 (0.88, 0.95)	0.92 (0.89, 0.95)***	0.87 (0.83, 0.92)	0.88 (0.84, 0.93)***
LDL‐C, mmol/L	2.94 (2.36, 3.55)	2.83 (2.29, 3.41)	2.74 (2.15, 3.33)***	3.02 (2.43, 3.63)	2.88 (2.30, 3.46)***
HDL‐C, mmol/L	1.29 (1.09, 1.52)	1.17 (1.00, 1.37)	1.11 (0.95, 1.32)***	1.36 (1.16, 1.58)	1.28 (1.10, 1.50)***
TC, mmol/L	5.05 (4.33, 5.78)	4.79 (4.13, 5.48)	4.70 (4.01, 5.44)**	5.18 (4.46, 5.92)	5.06 (4.33, 5.78)***
Triglyceride, mmol/L	1.37 (0.98, 1.97)	1.38 (0.98, 2.04)	1.56 (1.07, 2.33)***	1.32 (0.96, 1.89)	1.56 (1.11, 2.22)***
ALT, U/L	15.00 (11.00, 21.00)	16.00 (12.00, 23.00)	17.00 (12.00, 25.00)**	14.00 (11.00, 20.00)	15.00 (11.00, 21.00)***
AST, U/L	20.00 (17.00, 25.00)	21.00 (17.00, 25.00)	21.00 (17.00, 27.00)**	20.00 (17.00, 24.00)	21.00 (17.00, 26.00)***
GGT, U/L	21.00 (15.00, 32.00)	26.00 (18.00, 39.00)	28.00 (19.00, 45.00)***	19.00 (14.00, 27.00)	20.00 (15.00, 31.00)***
eGFR, ml/(min·1.73 m^2^)	89.79 (80.15100.63)	95.66 (82.62106.93)	90.08 (75.62103.04)***	88.81 (80.29, 98.01)	85.68 (76.42, 95.61)***
FBG, mmol/L	5.53 (5.10, 6.17)	5.64 (5.18, 6.37)	6.19 (5.38, 7.97)***	5.46 (5.08, 5.99)	5.66 (5.17, 6.57)***
PBG, mmol/L	7.39 (6.02, 9.68)	7.54 (6.00, 10.09)	9.30 (6.80, 13.92)***	7.18 (5.98, 9.11)	8.20 (6.45, 11.29)***
HbA1c, %		5.90 (5.60, 6.30)	6.20 (5.80, 7.20)***	5.90 (5.60, 6.20)	6.00 (5.70, 6.50)***
SBP, mmHg	129.33 (117.00, 144.00)	131.00 (119.33, 144.33)	141.33 (125.00, 156.92)***	127.00 (115.33, 141.00)	136.33 (120.67, 152.33)***
DBP, mmHg	76.67 (70.00, 84.00)	79.00 (72.00, 86.00)	81.00 (74.00, 90.67)***	75.33 (68.67, 82.08)	77.67 (70.33, 85.33)***
Drinking, (%)					
Never	26 118 (75.2)	4431 (48.0)	676 (53.8)***	17 812 (86.2)	3199 (89.6)***
Occasional	6332 (18.2)	3033 (32.8)	354 (28.2)***	2612 (12.6)	333 (9.3)***
Frequently	2282 (6.6)	1774 (19.2)	226 (18.0)***	242 (1.2)	40 (1.1)***
Smoking, (%)					
Never	29 835 (85.9)	5331 (57.7)	729 (58.0)	20 275 (98.1)	3500 (98.0)
Occasional	778 (2.2)	528 (5.7)	71 (5.7)	157 (0.8)	22 (0.6)
Frequently	4119 (11.9)	3379 (36.6)	456 (36.3)	234 (1.1)	50 (1.4)
CVD (%)					
Yes	1657 (4.8)	466 (5.0)	113 (9.0)***	827 (4.0)	251 (7.0)***
No	33 075 (95.2)	8772 (95.0)	1143 (91.0)***	19 839 (96.0)	3321 (93.0)***
Married/cohabitating, (%)					
Yes	30 691 (88.4)	8721 (94.4)	1159 (92.3)	17 918 (86.7)	2893 (81.0)***
No	4041 (11.6)	517 (5.6)	97 (7.7)	2748 (13.3)	679 (19.0)***
Occupation					
Worker	2471 (7.1)	1026 (11.1)	102 (8.1)***	1195 (5.8)	148 (4.1)***
Farmer	1303 (3.8)	348 (3.8)	62 (4.9)***	725 (3.5)	168 (4.7)***
Administrative staff	946 (2.6)	432 (4.7)	43 (3.4)***	407 (2.0)	64 (1.7)***
Merchant	2659 (7.7)	963 (10.4)	85 (6.8)***	1446 (7.0)	165 (4.6)***
Unemployed/housewife	4589 (13.2)	1324 (14.4))	128 (10.2)***	2678 (13.1)	459 (12.9)***
Retire	22 764 (65.5)	5145 (55.7)	836 (66.6)***	14 215 (68.8)	2568 (71.9)***
Education level					
Illiteracy	1533 (4.4)	189 (2.0)	35 (2.8)	995 (4.8)	314 (8.8)***
Primary school	4151 (12.0)	870 (9.4)	136 (10.8)	2553 (12.4)	592 (16.6)***
Secondary school	24 541 (70.7)	6269 (54.7)	429 (65.5)	15 076 (73.0)	2373 (66.4)***
Junior college or above	4507 (13.0)	1910 (20.7)	262 (20.9)	2042 (9.9)	293 (8.2)***

*Note*: Data were mean ± SD or median (interquartile range) for skewed variables or numbers (proportions) for categorical variables.

Abbreviations: ALT, alanine aminotransferase; AST, aspartate aminotransferase; BMI, body mass index; CVD, cardiovascular disease; CVAI, Chinese Visceral Adiposity Index; DBP, diastolic blood pressure; eGFR, estimated glomerular filtration rate; FBG, fasting blood glucose; GGT, gamma‐glutamyl transferase; HbA1C, hemoglobin A1; HDL‐C, high‐density lipoprotein cholesterol; LDL‐C, low‐density lipoprotein cholesterol; PBG, post‐load blood glucose; SBP, systolic blood pressure; TC, total cholesterol; UACR, urinary albumin to creatinine ratio; WC, waist circumference; WHR, waist‐to‐hip ratio.

** *p* < .01;

*** *p* < .001.

### 3.2 The association between CVAI and increased UACR in the male subjects

Table 3 shows the association between variables and increased UACR (UACR ≥30 mg/g) in male subjects. In model 5, after adjusting for all confounders, CVAI (Q4: odds ratio [OR]: 1.24, 95% confidence interval [CI]: 1.02–1.50), WC (Q4: OR: 1.21, 95% CI: 1.00–1.48) and triglyceride (Q4: OR: 1.18, 95% CI: 0.97–1.44) were significantly correlated with UACR, whereas WHR (Q4: OR: 1.06, 95% CI: 0.88–1.28) and BMI (Q4: OR: 1.10, 95% CI: 0.91–1.33) were uncorrelated with UACR.

**TABLE 3:** Association of CVAI, BMI, WC, WHR, and triglyceride with increased UACR in male participants.

Variable	Module 0 OR (95% CI)	Module 1 OR (95% CI)	Module 2 OR (95% CI)	Module 3 OR (95% CI)	Module 4 OR (95% CI)	Module 5 OR (95% CI)
CVAI						
Q1	1	1	1	1	1	1
Q2	1.17 (0.97, 1.41)	1.07 (0.89, 1.30)	1.07 (0.88, 1.29)	1.00 (0.83, 1.21)	0.90 (0.74, 1.09)	0.86 (0.70, 1.04)
Q3	**1.40 (1.17, 1.67)** ***	**1.25 (1.04, 1.51)** *	**1.26 (1.04, 1.51)** *	1.14 (0.95, 1.38)	0.98 (0.81, 1.18)	0.89 (0.73, 1.08)
Q4	**2.16 (1.82, 2.55)** ***	**1.95 (1.63, 2.33)** ***	**1.93 (1.61, 2.31)** ***	**1.71 (1.42, 2.06)** ***	**1.38 (1.15, 1.67)** **	**1.24 (1.02, 1.50)** *
WHR						
Q1	1	1	1	1	1	1
Q2	1.13 (0.94, 1.34)	1.11 (0.92, 1.32)	1.10 (0.92, 1.32)	1.06 (0.89, 1.28)	0.98 (0.81, 1.17)	0.94 (0.78, 1.13)
Q3	**1.25 (1.05, 1.48)** *	**1.26 (1.05, 1.51)** *	**1.25 (1.05, 1.50)** *	1.16 (0.97, 1.39)	1.03 (0.85, 1.25)	0.92 (0.77, 1.11)
Q4	**1.50 (1.27, 1.77)** ***	**1.52 (1.27, 1.81)** ***	**1.50 (1.26, 1.79)** ***	**1.39 (1.16, 1.67)** ***	**1.22 (1.01, 1.46)** *	1.06 (0.88, 1.28)
WC						
Q1	1	1	1	1	1	1
Q2	1.06 (0.91, 1.30)	1.03 (0.85, 1.23)	1.03 (0.85, 1.23)	0.97 (0.80, 1.16)	0.89 (0.74, 1.07)	0.84 (0.70, 1.02)
Q3	**1.31 (1.11, 1.55)** *	**1.28 (1.08, 1.52)** **	**1.28 (1.07, 1.52)** **	1.17 (0.98, 1.40)	1.01 (0.84, 1.21)	0.93 (0.77, 1.11)
Q4	**1.78 (1.50, 2.11)** ***	**1.84 (1.54, 2.20)** ***	**1.83 (1.52, 2.19)** ***	**1.63 (1.36, 1.96)** ***	**1.34 (1.11, 1.62)** *	1.21 (1.00, 1.48)
BMI						
Q1	1	1	1	1	1	1
Q2	0.99 (0.83, 1.18)	0.98 (0.82, 1.17)	0.98 (0.82, 1.17)	0.93 (0.77, 1.11)	0.81 (0.67, 0.97)	0.77 (0.64, 0.93)
Q3	1.12 (0.95, 1.33)	1.17 (0.98, 1.39)	1.16 (0.97, 1.39)	1.07 (0.99, 1.28)	0.88 (0.73, 1.06)	0.82 (0.68, 0.98)
Q4	**1.51 (1.29, 1.78)** ***	**1.72 (1.45, 2.05)** ***	**1.71 (1.44, 2.03)** ***	**1.52 (1.28, 1.81)** ***	1.17 (0.98, 1.41)	1.10 (0.91, 1.33)
Triglyceride						
Q1	1	1	1	1	1	1
Q2	1.04 (0.87, 1.25)	0.98 (0.82, 1.18)	0.99 (0.82, 1.19)	0.95 (0.79, 1.14)	0.90 (0.75, 1.09)	0.87 (0.72, 1.06)
Q3	**1.39 (1.17, 1.65)** ***	**1.33 (1.12, 1.59)** **	**1.34 (1.12, 1.60)** **	**1.23 (1.02, 1.47)** *	1.14 (0.95, 1.37)	1.07 (0.88, 1.29)
Q4	**1.58 (1.33, 1.86)** ***	**1.66 (1.40, 1.96)** ***	**1.66 (1.40, 1.98)** ***	**1.44 (1.20, 1.74)** ***	**1.29 (1.07, 1.57)** **	1.18 (0.97, 1.44)

*Note*: Model 1 was adjusted for age and center. Model 2 was additionally adjusted for occupation, current drinking habits, and CVD history. Model 3 was additionally adjusted for ALT, GGT, eGFR, and total cholesterol. Model 4 was additionally adjusted for SBP and DBP. Model 5 was additionally adjusted for HbA1c, FBG, and PBG.

Abbreviations: ALT, alanine aminotransferase; AST, aspartate aminotransferase; BMI, body mass index; CI, confidence interval; CVAI, Chinese Visceral Adiposity Index; CVD, cardiovascular disease; DBP, diastolic blood pressure; eGFR, estimated glomerular filtration rate; FBG, fasting blood glucose; GGT, gamma‐glutamyl transferase; HbA1C, hemoglobin A1; HDL‐C, high‐density lipoprotein cholesterol; LDL‐C, low‐density lipoprotein cholesterol; OR, odds ratio; PBG, post‐load blood glucose; SBP, systolic blood pressure; UACR, urinary albumin to creatinine ratio; WC, waist circumference; WHR, waist‐to‐hip ratio. The bold values represents *p* < 0.05.

* *p* < 0.05;

** *p* < 0.01;

*** *p* < 0.001.

### 3.3 The association between CVAI and increased UACR in the female subjects

In model 5 (Table 4), after adjusting for all confounders, only triglyceride (Q4: OR: 1.18, 95% CI: 1.04–1.33) and CVAI (Q4: OR: 1.16, 95% CI: 1.01–1.34) were significantly correlated with UACR, however, WC (Q4: OR: 1.07, 95% CI 0.95–1.21), WHR (Q4: OR: 0.94, 95% CI: 0.84–1.07), BMI (Q4: OR: 1.05, 95% CI: 0.94–1.18), and UACR were not correlated.

**TABLE 4:** Association of CVAI, BMI, WC, WHR, and triglyceride with increased UACR in female participants.

Variable	Module 0 OR (95% CI)	Module 1 OR (95% CI)	Module 2 OR (95% CI)	Module 3 OR (95% CI)	Module 4 OR (95% CI)	Module 5 OR (95% CI)
CVAI						
Q1	1	1	1	1	1	1
Q2	**1.24 (1.11, 1.40)** ***	1.10 (0.98, 1.24)	1.11 (0.99, 1.26)	1.09 (0.97, 1.23)	0.98 (0.86, 1.11)	0.94 (0.83, 1.07)
Q3	**1.71 (1.53, 1.91)** ***	**1.33 (1.18, 1.50)** ***	**1.35 (1.19, 1.52)** ***	**1.30 (1.15, 1.47)** ***	1.06 (0.94, 1.20)	0.98 (0.86, 1.11)
Q4	**2.76 (2.49, 3.07)** ***	**1.90 (1.67, 2.16)** ***	**1.89 (1.66, 2.15)** ***	**1.79 (1.57, 2.04)** ***	**1.33 (1.16, 1.53)** ***	**1.16 (1.01, 1.34)** *
WHR						
Q1	1	1	1	1	1	1
Q2	**1.14 (1.03, 1.27)** **	1.06 (0.95, 1.18)	1.05 (0.94, 1.17)	1.04 (0.93, 1.16)	0.96 (0.86, 1.08)	0.92 (0.83, 1.03)
Q3	**1.24 (1.12, 1.37)** ***	**1.19 (1.06, 1.33)** *	**1.17 (1.05, 1.31)** *	**1.15 (1.03, 1.28)** *	1.02 (0.91, 1.14)	0.95 (0.84, 1.06)
Q4	**1.38 (1.25, 1.53)** ***	**1.29 (1.14, 1.44)** ***	**1.265 (1.12, 1.41)** ***	**1.22 (1.06, 1.37)** **	1.05 (0.93, 1.18)	0.94 (0.84, 1.07)
WC						
Q1	1	1	1	1	1	1
Q2	**1.18 (1.06, 1.31)** *	1.10 (0.98, 1.23)	1.10 (0.98, 1.22)	1.08 (0.97, 1.21)	0.97 (0.87, 1.09)	0.95 (0.85, 1.06)
Q3	**1.33 (1.20, 1.47)** ***	**1.20 (1.08, 1.34)** *	**1.19 (1.06, 1.32)** *	**1.16 (1.04, 1.30)** **	0.99 (0.89, 1.11)	0.95 (0.85, 1.06)
Q4	**1.65 (1.49, 1.83)** ***	**1.58 (1.38, 1.73)** ***	**1.52 (1.36, 1.70)** ***	**1.47 (1.31, 1.64)** ***	1.17 (1.04, 1.32)	1.07 (0.95, 1.21)
BMI						
Q1	1	1	1	1	1	1
Q2	1.06 (0.96, 1.18)	1.10 (0.99, 1.23)	1.10 (0.99, 1.23)	1.09 (0.97, 1.21)	0.99 (0.88, 1.10)	0.96 (0.86, 1.07)
Q3	**1.13 (1.02, 1.25)** *	**1.17 (1.05, 1.31)** *	**1.17 (1.05, 1.30)** *	**1.14 (1.02, 1.27)** *	0.97 (0.86, 1.08)	0.92 (0.82, 1.02)
Q4	**1.37 (1.24, 1.51)** ***	**1.51 (1.36, 1.68)** ***	**1.49 (1.34, 1.66)** ***	**1.44 (1.29, 1.60)** ***	**1.14 (1.01, 1.27)** *	1.05 (0.94, 1.18)
Triglyceride						
Q1	1	1	1	1	1	1
Q2	**1.29 (1.16, 1.44)** ***	1.08 (0.97, 1.22)	1.10 (0.98, 1.23)	1.10 (0.98, 1.24)	1.02 (0.91, 1.15)	1.00 (0.87, 1.12)
Q3	**1.60 (1.44, 1.74)** ***	**1.19 (1.07, 1.34)** *	**1.21 (1.08, 1.35)** *	**1.21 (1.08, 1.36)** **	1.09 (0.97, 1.23)	1.03 (0.91, 1.16)
Q4	**2.14 (1.93, 2.37)** ***	**1.55 (1.40, 1.73)** ***	**1.57 (1.41, 1.75)** ***	**1.55 (1.38, 1.74)** ***	**1.31 (1.16, 1.48)** ***	**1.18 (1.04, 1.33)** **

*Note*: Model 1 was adjusted for age and center. Model 2 was additionally adjusted for occupation, current drinking habits, and CVD history. Model 3 was additionally adjusted for ALT, GGT, eGFR, and total cholesterol. Model 4 was additionally adjusted for SBP and DBP. Model 5 was additionally adjusted for HbA1c, FBG, and PBG.

Abbreviations: ALT, alanine aminotransferase; AST, aspartate aminotransferase; BMI, body mass index; CI, confidence interval; CVAI, Chinese Visceral Adiposity Index; CVD, cardiovascular disease; DBP, diastolic blood pressure; eGFR, estimated glomerular filtration rate; FBG, fasting blood glucose; GGT, gamma‐glutamyl transferase; HbA1C, hemoglobin A1; HDL‐C, high‐density lipoprotein cholesterol; LDL‐C, low‐density lipoprotein cholesterol; OR, odds ratio; PBG, post‐load blood glucose; SBP, systolic blood pressure; UACR, urinary albumin to creatinine ratio; WC, waist circumference; WHR, waist‐to‐hip ratio. The bold values represents *p* < 0.05.

* *p* < .05;

** *p* < .01;

*** *p* < .001.

### 3.4 Stratification analysis

In Table 5, in male or female normotensive participants, CVAI, BMI, WC, WHR, and triglycerides were not associated with UACR. In male hypertensive participants, CVAI (Q4: OR: 1.54, 95% CI: 1.17–2.01), BMI (Q4: OR: 1.33,95% CI: 1.03–1.72), WC (Q4: OR: 1.43, 95% CI: 1.10–1.85), and triglycerides (Q4: OR: 1.30, 95% CI: 1.01–1.67) were significantly associated with UACR, whereas WHR (Q4: OR: 1.20, 95% CI: 0.94–1.54) was not associated with UACR. In female hypertensive participants, CVAI (Q4: OR: 1.41, 95% CI: 1.12–1.78), triglycerides (Q4: OR: 1.32, 95% CI: 1.11–1.58), and WC (Q4: OR: 1.25, 95% CI: 1.04–1.49) were significantly associated with UACR, whereas BMI (Q4: OR: 1.15, 95% CI: 0.97–1.36) and WHR (Q4: OR: 1.03, 95% CI: 0.86–1.23) were not associated with UACR.

**TABLE 5:** Association of CVAI, BMI, WC, WHR, and triglyceride with increased UACR in hypertensive participants or normotensive participants.

Variable	Normal blood pressure				Hypertension			
	Q1	Q2	Q3	Q4	Q1	Q2	Q3	Q4
Male								
CVAI	1	0.77 (0.59, 1.02)	0.76 (0.57, 1.02)	1.00 (0.74, 1.35)	1	1.03 (0.77, 1.38)	1.09 (0.82, 1.44)	**1.54 (1.17, 2.01)** **
BMI	1	0.77 (0.59, 0.99)	0.73 (0.55, 0.98)	0.90 (0.67, 1.22)	1	0.87 (0.66, 1.14)	0.96 (0.74, 1.25)	**1.33 (1.03, 1.72)** *
WC	1	0.87 (0.67, 1.14)	0.71 (0.53, 0.94)	1.03 (0.76, 1.41)	1	0.89 (0.67, 1.17)	1.17 (0.91, 1.51)	**1.43 (1.10, 1.85)** **
WHR	1	0.88 (0.67, 1.15)	0.91 (0.68, 1.22)	0.96 (0.71, 1.29)	1	1.09 (0.84, 1.41)	1.04 (0.81, 1.34)	1.20 (0.94, 1.54)
Triglyceride	1	0.83 (0.62, 1.11)	0.94 (0.70, 1.26)	0.99 (0.72, 1.36)	1	0.92 (0.72, 1.19)	1.14 (0.89, 1.45)	**1.30 (1.01, 1.67)** *
Female								
CVAI	1	0.96 (0.83, 1.11)	0.92 (0.78, 1.08)	1.12 (0.92, 1.35)	1	1.00 (0.79, 1.27)	1.17 (0.93, 1.47)	**1.41 (1.12, 1.78)** **
BMI	1	0.99 (0.87, 1.15)	0.91 (0.79, 1.06)	1.11 (0.95, 1.30)	1	1.01 (0.84, 1.21)	1.02 (0.86, 1.21)	1.15 (0.97, 1.36)
WC	1	0.97 (0.84, 1.11)	0.94 (0.81, 1.09)	1.03 (0.87, 1.22)	1	1.02 (0.84, 1.23)	1.09 (0.91, 1.31)	1.25 (1.04, 1.49)
WHR	1	0.93 (0.81, 1.07)	0.96 (0.83, 1.12)	0.96 (0.81, 1.14)	1	1.01 (0.84, 1.21)	1.02 (0.85, 1.21)	1.03 (0.86, 1.23)
Triglyceride	1	0.98 (0.84, 1.14)	0.94 (0.80, 1.11)	1.12 (0.95, 1.33)	1	1.07 (0.89, 1.29)	1.18 (0.99, 1.42)	**1.32 (1.11, 1.58)** **

*Note*: Adjusted for age, center, occupation, current drinking habits, CVD history, ALT, GGT, eGFR, total cholesterol, HbA1c, FBG, and PBG.

Abbreviations: ALT, alanine aminotransferase; BMI, body mass index; CVAI, Chinese Visceral Adiposity Index; CVD, cardiovascular disease; eGFR, estimated glomerular filtration rate; FBG, fasting blood glucose; GGT, gamma‐glutamyl transferase; HbA1C, hemoglobin A1; PBG, post‐load blood glucose; WC, waist circumference; WHR, waist‐to‐hip ratio. The bold values represents *p* < 0.05.

* *p* < .05;

** *p* < .01;

*** *p* < .001.

In Table 6, in male normoglycemic participants, CVAI, BMI, WC, WHR, and triglycerides were not associated with UACR. Among female normoglycemic participants, only triglycerides were significantly associated with UACR, whereas CVAI, BMI, WC, and WHR were not correlated with UACR. In male hyperglycemic participants, CVAI (Q4: OR: 1.37, 95% CI: 1.10–1.72) and WC (Q4: OR: 1.32, 95% CI: 1.04–1.68) were significantly associated with UACR, whereas BMI (Q4: OR: 1.13, 95% CI: 0.90–1.43), WHR (Q4: OR: 1.19, 95% CI:0.95–1.49), and triglycerides (Q4: OR: 1.27, 95% CI: 0.99–1.62) were not. In female hyperglycemic participants, CVAI (Q4: OR: 1.38, 95% CI: 1.15–1.66), BMI (Q4: OR: 1.18, 95% CI: 1.01–1.38), and WC (Q4: OR: 1.27, 95% CI: 1.08–1.50) were significantly associated with UACR, whereas WHR (Q4: OR: 1.13, 95% CI: 0.96–1.32) and triglycerides (Q4: OR: 1.13, 95% CI: 0.95–1.33) were not.

**TABLE 6:** Association of CVAI, BMI, WC, WHR, and triglyceride with increased UACR in normoglycemic participants or in participants with diabetes or prediabetes.

Variable	Normal blood glucose				Diabetes or prediabetes			
	Q1	Q2	Q3	Q4	Q1	Q2	Q3	Q4
Male								
CVAI	1	0.73 (0.51, 1.04)	0.87 (0.60, 1.26)	1.30 (0.89, 1.89)	1	0.98 (0.77, 1.24)	0.99 (0.79, 1.25)	**1.37 (1.10, 1.72)** **
BMI	1	0.61 (0.44, 0.83)	0.82 (0.60, 1.11)	1.15 (0.84, 1.56)	1	0.91 (0.72115)	0.88 (0.69, 1.11)	1.13 (0.90, 1.43)
WC	1	0.66 (0.48, 0.91)	0.95 (0.70, 1.29)	1.32 (0.96, 1.82)	1	1.03 (0.81, 1.31)	1.02 (0.81, 1.28)	**1.32 (1.04, 1.68)** *
WHR	1	0.95 (0.70, 1.29)	0.87 (0.63, 1.20)	1.19 (0.86, 1.63)	1	0.97 (0.77, 1.23)	1.06 (0.84, 1.33)	1.19 (0.95, 1.49)
Triglyceride	1	0.84 (0.63, 1.15)	1.13 (0.83, 1.54)	1.10 (0.78, 1.55)	1	0.91 (0.72, 1.17)	1.10 (0.87, 1.40)	1.27 (0.99, 1.62)
Female								
CVAI	1	0.85 (0.71, 1.02)	0.96 (0.79, 1.16)	1.08 (0.86, 1.39)	1	1.07 (0.89, 1.28)	1.09 (0.92, 1.30)	**1.38 (1.15, 1.66)** **
BMI	1	0.92 (0.79, 1.08)	0.87 (0.74, 1.02)	0.96 (0.81, 1.14)	1	1.03 (0.88, 1.22)	1.01 (0.86, 1.18)	**1.18 (1.01, 1.38)** *
WC	1	0.91 (0.77, 1.06)	0.87 (0.74, 1.03)	0.94 (0.78, 1.13)	1	1.02 (0.86, 1.20)	1.08 (0.92, 1.26)	**1.27 (1.08, 1.50)** *
WHR	1	0.96 (0.82, 1.15)	0.97 (0.83, 1.15)	0.84 (0.70, 1.02)	1	0.95 (0.81, 1.11)	1.01 (0.87, 1.18)	1.13 (0.96, 1.32)
Triglyceride	1	1.01 (0.86, 1.18)	1.08 (0.91, 1.27)	**1.26 (1.05, 1.51)** *	1	0.96 (0.82, 1.14)	0.97 (0.81, 1.15)	1.13 (0.95, 1.33)

*Note*: Adjusted for age, center, occupation, current drinking habits, CVD history, ALT, GGT, eGFR, total cholesterol, SBP, and DBP.

Abbreviations: ALT, alanine aminotransferase; BMI, body mass index; CVAI, Chinese Visceral Adiposity Index; CVD, cardiovascular disease; DBP, diastolic blood pressure; eGFR, estimated glomerular filtration rate; FBG, fasting blood glucose; GGT, gamma‐glutamyl transferase; SBP, systolic blood pressure; UACR, urinary albumin to creatinine ratio; WC, waist circumference; WHR, waist‐to‐hip ratio. The bold values represents *p* < 0.05.

* *p* < .05;

** *p* < .01;

*** *p* < .001.

## 4 DISCUSSION

As far as we know, this is the first study to explore the relationship between CVAI and UACR in a large sample, multicenter Chinese general population. The principal discoveries of this study indicated that CVAI was significantly associated with increased UACR in male and female Chinese community adults. In men, CVAI and WC had similar ability to discriminate albuminuria, and they were stronger than BMI, WHR, and triglycerides in discriminating albuminuria. In women, CVAI and triglycerides had similar ability to discriminate albuminuria, and they were stronger than BMI, WHR, and WC in discriminating albuminuria. Besides, CVAI was more strongly associated with elevated UACR than traditional indicators of obesity and triglycerides in men or women with hypertension or hyperglycemia. Therefore, for people with hypertension or hyperglycemia, it is important to monitor CVAI and prevent albuminuria in time.

A robust body of literature has uncovered that visceral obesity is highly associated with renal dysfunction and microalbuminuria. Chung's study suggested that patients with type 2 diabetes who had more visceral adipose were more likely to develop diabetic nephropathy.^14^ A research by Framingham Heart Study reported that abdominal visceral adiposity was more closely related to microalbuminuria than subcutaneous adipose tissue.^12^ Kim et al declared that the visceral adipose tissue cross‐sectional area measured by CT was strongly connected with albuminuria among the healthy Asian populace.^32^ A 4‐year prospective cohort investigation in Korea proved that research subjects with higher basic line visceral adipose had several times the incremental danger of albuminuria.^16^ However, given the inconveniences of imaging techniques, a reliable obesity index is vital. The traditional obesity index, such as BMI could not distinguish whole‐length adipose distribution, meanwhile, it might overpredict the hazard of illnesses among individuals with lots of muscle mass.^33^ WC and WHR are advantageous to use in clinical practice, but the correlation between WC or WHR and subcutaneous adiposity was stronger than that between WC or WHR and visceral adiposity.^34^ In addition, WC and WHR do not reflect the effect of height on disease risk. CVAI contains BMI, WC, age, and lipids, and CVAI is independent of somatotype.^19^

Studies on the relationship between CVAI and UACR are scarce. Lu et al reported that CVAI had the strongest association with UACR among the abdominal obesity indices.^21^ The previous study concentrated on diabetics only, whereas the current study was large‐scale, multicenter, and publicly engaged. The dependent variable UACR in their study was not classified according to clinical definitions, in the present study; however, UACR was classified according to clinical definitions, Besides, in our study, the relationship between increased UACR and CVAI was stratified based on blood pressure and blood glucose. The present study could be served as a supplement to the previous study, and the present study demonstrated that CVAI, as an obesity indicator that could predict albuminuria, was not only suitable for people with diabetes but also for the general population. This is important on account that clinically some people only have albuminuria without diabetes or kidney disease.^35^ The conclusions of the current study provide a way for the public that they could recognize albuminuria risk by elevated CVAI values and timely change abnormal adiposity distribution to postpone or refrain from the occurrence of albuminuria.

The relationship between CVAI and increased UACR has a slight gender difference. A study on 35 751 Chinese population found that triglyceride and UACR were significantly correlated only in women, whereas there was no correlation between triglyceride and UACR in men.^36^ A study of 35 848 Chinese population found that residues of triglyceride‐rich lipoproteins were associated with UACR only in the female population but not in the male population.^37^ Hokanson conducted a meta‐analysis based on the Framingham Heart Study, and he found that elevated triglyceride was more important for women than for men.^38^ In fact, although elevated triglyceride is proportional to the severity of albuminuria, when combined with overweight or obesity, the renal function is more likely to be damaged.^39^ We speculate that CVAI is highly associated with increased UACR because it is an indicator that includes both lipids and obesity measures. In the current study, although the OR value of CVAI was similar to that of triglyceride in the female population, the OR value of CVAI was higher than that of triglycerides in the male population, so we consider that CVAI could better predict UACR than triglyceride. Besides, previous studies have shown that women are more likely to have visceral obesity,^40^ and there is a greater association between visceral obesity and adverse metabolic outcomes in women,^41^ so we believe that women should be alert to the risk of albuminuria from visceral adiposity even if they are not overweight.

It is worth noting that after adjusting for SBP and DBP in model 4 and HbA1c, FBG, and PBG in model 5, the correlation between CVAI and UACR was attenuated, but still present. This indicates that abnormal metabolism might raise the risk of albuminuria. Many previous studies have shown important relationships between albuminuria, hyperglycemia, and hypertension,^42^ which could in part account for our findings. In the stratified analysis of the current study, we found that CVAI and UACR were significantly correlated in men and women with prediabetes, diabetes, and hypertension; thus we speculated that alterations in blood pressure and blood glucose were involved in the relationship between CVAI and albuminuria. Furthermore, some authors have suggested that excessive leptinemia and lipocalin deficiency in viscerally obese patients may directly stimulate the renin‐angiotensin‐aldosterone system,^43^ which would lead to albuminuria and hypertension,^43^ and this could partly explain the stronger correlation between CVAI and UACR in the hypertensive population because hypertension and albuminuria might occur together in viscerally obese patients. Besides, we found that in populations with hypertension or hyperglycemia, CVAI was better at discriminating elevated UACR than traditional obesity indices and triglycerides. Consequently, for individuals with abnormal blood pressure or blood glucose, reducing visceral adipose is equally critical as lowering blood pressure and glucose, and it is simple and feasible to monitor elevated CVAI to identify excess visceral adiposity and albuminuria risk.

The mechanism of the association between visceral adipose and albuminuria is worth discussing. (1) Adiponectin decreases as visceral fat increases, and previous studies report an inverse association between adiponectin and proteinuria.^12^ (2) Leptin levels increase with increased visceral fat. Leptin causes albuminuria via inducing glomerular endothelial cell proliferation, increasing glomerular transforming growth factor‐β1 (TGF‐b1) expression, and increasing synthesis of type I collagen and type IV messenger RNA (mRNA).^43^ (3) The secretion of free fatty acids and interleukin 6 (IL‐6) increases with the volume of visceral adipose tissue, which in turn contributes to macrophage infiltration and overproduction of tumor necrosis factor α (TNF‐α), resulting in chronic inflammation and renal failure.^44^ (4) Superfluous visceral adipose compresses the kidneys and raises intrarenal pressure.^45^ (5) Superabundant visceral fat stimulates juxtaglomerular cells to secrete renin.^45^ (6) A slight increase in plasma aldosterone in viscerally obese patients leads to salt and water retention, which in turn leads to albuminuria.^46^

Thinking over the strong association between visceral adipose, albuminuria, and CVD, individuals with excess visceral fat ought to change their fat distribution rather than only weight loss. Regular exercise for 8 weeks to 1 year could reduce total, subcutaneous, and visceral adipose, and for exercise patterns, high‐intensity interval training is more targeted to reduce visceral adipose than moderate intensity continuous training.^47^ Besides, a combination of a low‐calorie diet and regular exercise is more effective in improving adipose distribution.^47^ Furthermore, when diet control and regular exercise are not effective, a combination of orlistat could also contribute to reducing excess visceral adipose tissue.^48^

The first advantage of this study was the large sample size, and the inspection is very detailed, therefore, the confounders could be fully adjusted. Second, the samples were community based, so they could represent the general population of China. Third, the relationship between CVAI and increased UACR was investigated in a general Chinese population, for which no previous studies have been conducted. Nevertheless, there were some limitations. First, the current study was cross‐sectional, thus, we could only draw conclusions about the association, not causation. Prospective cohort studies are needed in the future. Second, we did not measure UACR multiple times, but we limit urine collection to the first morning to minimize the impact of daytime activity.

## 5 CONCLUSION

Generally speaking, in the Chinese general population, CVAI was closely associated with increased UACR in both genders. Besides, at higher CVAI levels, the population with prediabetes, diabetes, or hypertension had a higher risk of albuminuria. We believe that CVAI could be a credible and convenient predictor of albuminuria and has guidance meaning to assist individuals to maintain healthy bodily form, especially for people with hyperglycemia or hypertension.

## FUNDING INFORMATION

The study is supported by Beijing Municipal Science and Technology Commission (Z201100005520014).

## CONFLICTS OF INTEREST

The authors report no conflicts of interest in this work.

## ETHICAL APPROVAL

The study protocol was approved by the Committee on Human Research at Rui‐Jin Hospital affiliated with the School of Medicine, Shanghai Jiao Tong University. Written informed consents were obtained from all participants before data collection.
